# Genomic and spatial epidemiology: lessons learned from SARS-CoV-2 pandemic

**DOI:** 10.1097/COH.0000000000000936

**Published:** 2025-04-01

**Authors:** Yangji Choi, David De Ridder, Gilbert Greub

**Affiliations:** a Institute of Microbiology, Lausanne University Hospital and University of Lausanne; b Group of Geospatial Molecular Epidemiology (GEOME), Laboratory for Biological Geochemistry (LGB), School of Architecture, Civil and Environmental Engineering (ENAC), École Polytechnique Fédérale de Lausanne (EPFL), Lausanne; c Group of Geographic Information Research and Analysis in Population Health (GIRAPH); d Faculty of Medicine, University of Geneva (UNIGE); e Division and Department of Primary Care Medicine, Geneva University Hospitals, Geneva; f Service of Infectious Diseases, Lausanne University Hospital and University of Lausanne, Lausanne, Switzerland

**Keywords:** Coronavirus, COVID-19, genomic epidemiology, interdisciplinarity, pandemics, public health, SARS-CoV-2, spatial epidemiology, temporospatial epidemiology, viral genomics

## Abstract

**Purpose of review:**

The SARS-CoV-2 pandemic presented unprecedented challenges, particularly in understanding its complex spatial transmission patterns. The high transmissibility of the virus led to frequent super-spreading events. These events demonstrated clear spatial clustering patterns, often tied to specific events that facilitated transmission. The uneven geographic distribution of medical resources and varying access to care amplified the impact of SARS-CoV-2. Asymptomatic cases further complicated the situation, as infected individuals could silently spread the virus before being identified.

Thus, this review examines how genomic and spatial epidemiology approaches can be integrated to answer some of the above-mentioned challenges. We first describe the methodological foundations of genomics and spatial epidemiology, detailing opportunities of their applications during the SARS-CoV-2 pandemic. We then present a novel interdisciplinary framework that combines these approaches to better guide public health interventions.

**Recent findings:**

During the pandemic, the genomic and spatial approaches were used to address key questions, including “how does the pathogen evolve and diversify?” and “how does the pathogen spread geographically?”. Genomic epidemiology allows researchers to identify viral lineages and new variants. Conversely, spatial epidemiology focused on geographic distribution of infections, analyzing how the virus spread. However, despite their complementary nature, these approaches were largely applied independently during the pandemic. This separation limited our collective ability to fully understand the complex relationships between viral evolution and geographic spread.

**Summary:**

While phylogeography has traditionally combined phylogenetic and geographic data to understand long-term evolutionary patterns across large areas, events such as the recent SARS-CoV-2 pandemic demand frameworks that can inform public health interventions through joint analysis of genomic and local-scale spatial data.

## INTRODUCTION

### Genomic and spatial epidemiology

#### Genomic approaches for epidemiology

The concept of precision public health – delivering the right intervention to the right population at the right time – emphasizes the need for integrating multiple data streams to enable targeted responses [1–4]. Genomics is one of the approach – along spatial epidemiology – that may be used to perform precision epidemiology. Genomics has become an indispensable public health tool, particularly during the SARS-CoV-2 pandemic due to the virus's high mutation rate and frequent emergence of new variants [5–7]. These mutations affected transmissibility [8,9^▪▪^], immune evasion [10,11], and vaccine efficacy [12], necessitating continuous genomic surveillance to guide public health responses.

Genomic approaches offer distinct advantages over other methods by providing definitive biological evidence about pathogens as the genome contains the most fundamental information such as evolution, transmissibility, drug resistance, and clinical outcomes [13]. Genomic data allows for high-resolution identification of viral mutations, from insertions and deletions (indels) to single nucleotide variants (SNVs). These sequences can be used as genomic fingerprints for rigorous comparisons and differentiations of viral genomes, allowing for identifying new strains and tracing transmission pathways. Genomic analysis can also confirm suspected transmission and uncover hidden clusters, such as superspreading events [14–16]. While RT-PCR assays can quickly detect known variants by targeting specific mutations, they cannot identify novel variants, recombinants, or emerging strains that contain previously undocumented genetic changes [17]. In contrast, whole genome sequencing (WGS), particularly using next-generation sequencing (NGS) technologies, remains the gold standard for variant identification, providing a complete picture of viral genomes, including unknown variants [18,19].

Genomic epidemiology typically begins with whole genome sequencing, which provides the basis for various subsequent analyses. The two primary NGS technologies are short-read sequencing (e.g., Illumina) and long-read sequencing (e.g., Oxford Nanopore Technologies and PacBio). Short-read sequencing offers high accuracy and high throughput but may introduce artifacts during computational assembly of overlapping short fragments. Long-read sequencing is faster, but has higher error rates, which have been improved with recent advances in error correction algorithms making it more suitable for rapid sequencing in outbreak [20]. 

**Box 1:**
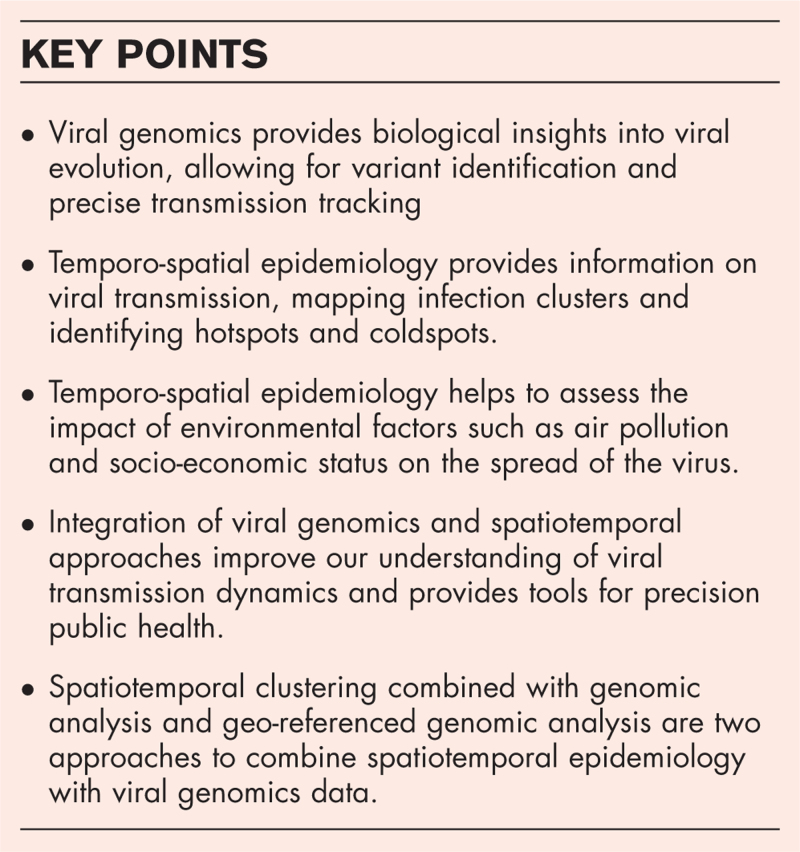
no caption available

**FIGURE 1:**
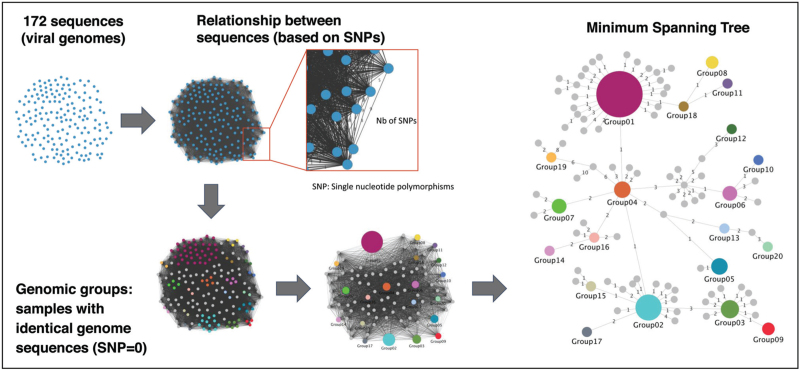
How to obtain a Minimum Spanning Tree ? Starting from 172 SARS-CoV-2 viral genomes, we first assessed the relationships between all genomes by calculating the number of SNPs between each pair of genomes. Then, all genomes with exactly the same sequence (SNP=0) are clustered together (with a circle larger when the number of samples in a given genomic cluster is higher). Then, we connected each genomic cluster with the closer clusters, that is, clusters with the lower number of SNPs. Thus, we finally obtained a Minimum Spanning Tree, that is, a tree connecting all nodes in a graph, in a way such that the sum of edge lengths is minimized.

After sequencing and quality assessment, viral genomes undergo three complementary analytical approaches. Genomic distance analysis measures the genetic distance between genomes based on SNVs, enabling identification of closely related cases that likely represent transmission clusters, with smaller distances indicating closer relationships. Phylogenetic analysis constructs trees to visualize evolutionary relationships and infer when variants emerged, providing insights into the temporal dynamics of epidemics. Finally, transmission network analysis maps direct transmission events, representing viral genomes (cases) as nodes and genetic relationships (transmission) as edges. This approach is efficient for capturing transmission clusters, including superspreading events, which is crucial for contact tracing and targeted interventions. Such transmission network analysis may be represented for example by a Minimum Spanning Tree (Fig. 1).

### Spatial approaches for epidemiology

During the SARS-CoV-2 pandemic, spatial approaches proved essential for understanding transmission dynamics when traditional methods like contact tracing were limited by the virus's high transmissibility and capacity for indirect transmission. Spatial methods were therefore particularly valuable for rapid identification of transmission patterns and hotspots, providing essential information for effective outbreak management.

Spatial and spatio-temporal epidemiology examines how diseases vary across both space and time, analyzing their relationships with demographic, environmental, and socioeconomic factors [21,22]. Given that disease spread is inherently spatial, analyzing spatial variation is crucial to understand transmission dynamics [23]. This field originated with spatial investigations, such as John Snow's map of cholera cases in 1854, which demonstrated the power of spatial analysis in understanding disease transmission [24]. More recently, Pavlovsky further developed this approach through three principles : diseases tend to be restricted geographically, spatial variation results from underlying physical or biological variation associated with pathogens and hosts, and mapping such conditions allows for prediction of current and future prevalence and risk [21,25]. Modern spatiotemporal methods have significantly advanced these foundational principles through modern Geographic Information Systems (GIS) and spatial statistics. GIS enable sophisticated spatial data integration and processing, visualization of spatially enabled epidemiological data, but also of diverse information layers, including population density, mobility patterns, and environmental features [26–28]. This layering of information through location also represents a cost-effective solution for missing socio-economic data when direct collection is difficult or costly [29]. While complex forms of spatial data exist and are valuable, GIS can leverage simpler location data such as routinely collected residential or work addresses. Through geocoding, these addresses are converted into geographic coordinates (i.e., longitude and latitude) [30], enabling GIS and quantitative spatial analysis, ranging from distance calculations between cases to sophisticated clustering methods [26,31].

These capabilities support multiple analytical approaches, from cases mapping and geographic correlation analysis that reveal spatial patterns and relationships between disease spread and environmental or socioeconomic factors, to comprehensive risk assessments that identify high-risk areas based on multiple data layers [21,28,32]. Cluster detection, using methods like spatial scan statistics, has emerged as a particularly powerful tool for identifying disease hotspots [17–19].

Spatiotemporal clustering represents an advanced approach by incorporating temporal dimensions. As defined by Knox [19], spatial clusters are “geographically bounded groups of occurrences of sufficient size and concentration to be unlikely to have occurred by chance.” These clusters help identify areas of locally elevated disease risk and physical proximity that may facilitate transmission [20]. Modern analytical methods implement this concept by using various techniques, from density-based approaches that identify clusters through point concentrations and connectivity, to scan statistics, which use moving circular windows to detect spatial clusters [21]. This approach extends to spatiotemporal analysis by expanding the window into a cylinder, enabling detection of clusters that are similar in both space and time [22]. Each method offers different advantages for detecting disease hotspots depending on the spatial and temporal characteristics of the outbreak.

**FIGURE 2:**
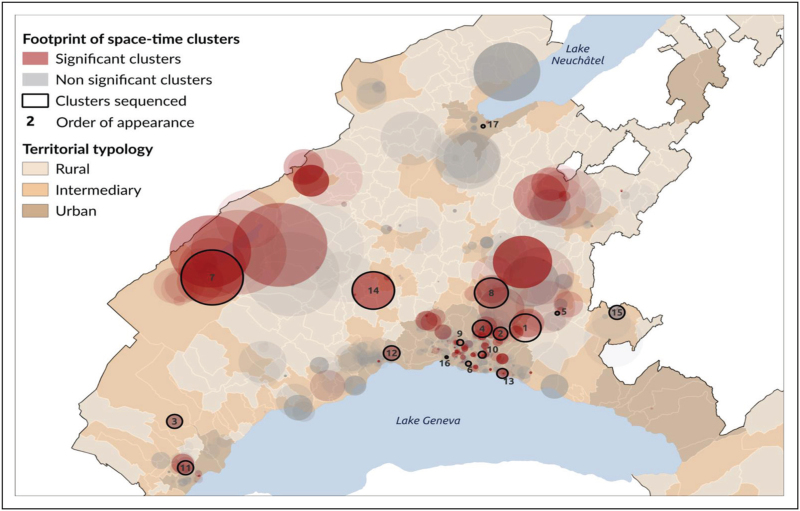
Example of spatio-temporal analysis. The figure shows different clusters numbered in order of appearance. The size of the cluster corresponds to the proportion of cases (infections) detected in a given place during a defined period according to the population density (number of persons susceptible to get infected). This example shows that we can layer the information on the cluster with geographical data, for example here a variable (urban, intermediate versus rural), shown with different shades of brown that correlate with population density and hence with cluster size. Please note that some circles, despite being large due to a low population density, did not correspond to statistically robust clusters since the number of observed cases per population density is too low to reach statistical significance.

These integrated approaches have proven essential for infectious disease epidemiology, supporting evidence-based public health responses through improved understanding of disease spread patterns and environmental influences [12]. Fig. 2 shows an example of such a spatiotemporal analysis.

## SPATIO-GENOMIC EPIDEMIOLOGY: FROM SILOS TO SYNERGY

### Integration potential of spatio-genomics for data-driven analysis

Genomic and spatial epidemiology share fundamental characteristics that align with the concept of precision public health, an approach that uses the best available data to more effectively target interventions to those who need them most [33–35]. Just as precision medicine aims to provide the right treatment to the right patient at the right time, precision public health leverages high-resolution data to deliver the right intervention to the right population at the right time [4]. Three key features of genomic and spatial approaches enable this precision: high data resolution, quantitative analysis capabilities, and complementary epidemiological assumptions.

The first key feature is high-resolution data, which supports detailed and high-quality analysis. Advanced sequencing technologies provide whole genome sequences differentiated at the single nucleotide level, enabling precise identification of viral strains. Similarly, spatial approaches provide multiscale geographic precision, from individual-level coordinates through geocoded addresses to population-level patterns of mobility and environmental exposure, enabling targeted interventions at various spatial scales. This precision in both domains allows for detailed overlay analysis and hypothesis generation.

The second key feature is their inherently quantitative nature. Genomic data, particularly SNVs, enable calculation of genetic distances, pairwise similarity, and diversity. Such quantitative data are used to model phylogenetic trees and transmission networks, illustrating evolutionary and epidemiological relationships between cases. It also allows the estimation of critical epidemiological parameters such as viral population size, epidemic doubling time and basic reproduction number (Rt) [36]. In the spatial domain, coordinates enable mathematical representations of disease spread that can be analyzed at multiple scales, from individual transmission events to population-level dynamics. These quantitative spatial relationships can be directly compared with genetic distances, creating opportunities for integrated spatiotemporal and genomic analyses [37].

The third key feature is the ability of both methods to generate testable epidemiological hypotheses. Genomic analysis identifies sets of cases with similar or identical viral genomes (“genomic clusters”), which can be represented as a monophyletic group in a phylogenetic tree or as a large node in a transmission network. These patterns of genetic relatedness suggest that they may have originated from the same transmission event, with large genomic clusters indicating potential superspreading events. Complementarily, spatial relationships between cases suggest potential transmission pathways based on geographic proximity and connectivity patterns, through direct and indirect local contacts between individuals. Together, these approaches provide a framework for hypothesis generation about transmission dynamics, from local spread within households to larger-scale community transmission events.

### Complementary features of genomic and spatial approaches

While precision public health aims to deliver the right intervention to the right population at the right time, achieving this goal requires balancing precision with timeliness. Genomic epidemiology provides high biological precision, enabling variant-specific interventions and precise transmission chain identification. However, during the SARS-CoV-2 pandemic, typical WGS workflows required multiple days and remained expensive, creating a significant delay between case identification and genomic insights. For example, in Lausanne, we have done weekly analysis and time to results ranged from 5 to 12 days, since 1–7 days delay is due to the time from sampling to start of the analysis and since the whole analytical process takes typically 5 days, to have the viral RNA extracted, the library prepared, the sequencing done, and the genomes sequences quality-checked, biomedically validated and submitted to the various databases (such as GISAID). Furthermore, most genomic studies, with very few exceptions [38^▪^,^39^,40^▪^], relied on broad administrative divisions as geographic identifiers, limiting the spatial precision needed for targeted interventions [41–44]. Indeed, administrative units are often too broad to accurately reflect real-world transmission dynamics since the virus is not constrained by jurisdictional boundaries.

In contrast, spatial epidemiology offers a different dimension of precision through rapid geographic targeting of interventions. Within hours of case reporting, spatial analyses can identify potential hotspots and risk factors across multiple geographic scales, enabling swift public health responses. However, when based solely on PCR-confirmed cases, these analyses cannot distinguish between variants or definitively establish transmission links, limiting intervention specificity.

The complementary nature of these limitations suggests an opportunity to optimize precision public health responses. While genomic methods provide precision in “what” (variant) and “who” (transmission links), spatial methods deliver precision in “where” and “when” interventions should occur. Integrating these approaches could help achieve the core aim of precision public health: delivering precisely targeted interventions with optimal timing.

### Spatio-genomic frameworks: case studies on SARS-CoV-2 pandemic

We present two frameworks developed during the SARS-CoV-2 pandemic and published in previous studies that demonstrate how integrating genomic and spatial epidemiology can complement each other to enhance precision public health responses.

#### Spatiotemporal clustering and genomics

The first framework combines spatiotemporal clustering with targeted genomic sequencing to investigate SARS-CoV-2 infection clusters and improve epidemic surveillance efficiency. Applied during the first epidemic wave in the canton of Vaud, Switzerland, this approach first identified statistically significant spatiotemporal clusters from PCR testing data (Fig. 2). These clusters then guided strategic whole genome sequencing efforts, enabling cost-effective genomic surveillance of potential transmission events.

The sequenced genomes were then analyzed using phylogenetic and network analysis methods (such as Minimum spanning tree, see Fig. 1). The phylogenetic analysis helped to establish evolutionary relationships between the virus strains that formed the same or different clusters. Genetic similarity within clusters, measured by the Jaccard index, was higher in rural areas and at the onset or lockdown period. In addition, network analysis identified two superspreading events characterized by two distinct genetic mutations, which originated from two major outbreaks in neighboring countries, France and Italy, respectively.

This approach identified key moments in the early outbreak, including introduction and superspreading events, suggesting prioritizing sequencing efforts based on spatiotemporal clusters to optimize resource allocation and surveillance efficiency.

#### Georeferenced genomic analysis

The second framework, applied later in the pandemic, integrates geographic data with genomic data to provide a comprehensive view of variant-specific transmission dynamics. By geocoding sequenced cases to residential locations, this approach revealed how different SARS-CoV-2 variants exhibited distinct spatial behaviors. Notably, the Omicron variant showed a greater spatial dispersion of genomic clusters compared to Alpha and Delta, indicating high transmission capacity and stochasticity. To further investigate local transmission, genetic diversity was calculated using genomic data, focusing specifically on neighborhood and household cases identified by geographic coordinates. During the invasion of the Omicron variant, genetic diversity within both neighborhood and household cases increased significantly, suggesting the ability of the variant to spread rapidly in a localized environment.

#### Geographical visualization of genomic data

Both frameworks leverage GIS-based visualization to translate complex spatio-genomic patterns into actionable insights. This approach aligns with one of the five key objectives outlined by WHO in the “Global Strategy for Genomic Surveillance of Pathogens of Pandemic and Epidemic Potential (2022–2032)” [45]. Notably, its first objective is to “improve access to tools for better geographic representation”. Beyond simple mapping, these visualizations reveal patterns of genetic diversity within spatiotemporal clusters and track variant-specific spread patterns, demonstrating how integrated spatio-genomic approaches can enhance precision surveillance capabilities.

## CONCLUSION

The SARS-CoV-2 pandemic underscored how integrating genomic and spatial epidemiology can advance precision public health responses to better understand and manage infectious disease outbreaks. While genomic approaches provide biological precision in identifying variants and transmission chains, and spatial methods enable rapid and precise geographic targeting of interventions, their integration offers a more comprehensive and detailed understanding of transmission dynamics. This provides unique opportunities to optimize both the precision and timeliness of public health surveillance and response. Our frameworks show how this integration can enhance surveillance efficiency through targeted sequencing strategies and reveal variant-specific spatial behaviors. Moving forward, as we prepare for future epidemics, developing standardized approaches for spatio-genomic analysis will be crucial. Such integration embraces interdisciplinarity and transforms methodological silos into powerful synergies for effective, evidence-based public health decision-making, ultimately enabling more precise and effective public health interventions.

## Acknowledgements

*None.*

### Financial support and sponsorship

*None.*

### Conflicts of interest

*There are no conflicts of interest.*

## REFERENCES AND RECOMMENDED READING

Papers of particular interest, published within the annual period of review, have been highlighted as:

▪ of special interest
▪▪ of outstanding interest

## References

[1] Precision public health: what is it? [Accessed March 25, 2025]. https://blogs.cdc.gov/genomics/2018/05/15/precision-public-health-2/.

[2] Roberts MC Holt KE Del Fiol G. Precision public health in the era of genomics and big data. Nat Med 2024; 30:1865–1873. 38992127 10.1038/s41591-024-03098-0 PMC12017803

[3] Rasmussen SA Khoury MJ Del Rio C. Precision public health as a key tool in the COVID-19 response. JAMA 2020; 324:933–934. 32805001 10.1001/jama.2020.14992

[4] Khoury MJ Armstrong GL Bunnell RE. The intersection of genomics and big data with public health: opportunities for precision public health. PLoS Med 2020; 17:e1003373. 33119581 10.1371/journal.pmed.1003373 PMC7595300

[5] Bloom JD Beichman AC Neher RA Harris K. Evolution of the SARS-CoV-2 mutational spectrum. Mol Biol Evol 2023; 40:msad085. 37039557 10.1093/molbev/msad085 PMC10124870

[6] Alquraan L Alzoubi KH Rababa’h SY. Mutations of SARS-CoV-2 and their impact on disease diagnosis and severity. Inform Med Unlocked 2023; 39:101256. 37131549 10.1016/j.imu.2023.101256 PMC10127666

[7] Flores-Vega VR Monroy-Molina JV Jiménez-Hernández LE. SARS-CoV-2: evolution and emergence of new viral variants. Viruses 2022; 14:653. 35458383 10.3390/v14040653 PMC9025907

[8] Davies NG Abbott S Barnard RC. Estimated transmissibility and impact of SARS-CoV-2 lineage B.1.1. 7 in England. Science 2021; 372:1–9. 10.1126/science.abg3055 PMC8128288 33658326

[9▪▪] Carabelli AM Peacock TP Thorne LG. COVID-19 Genomics UK Consortium, *et al.* SARS-CoV-2 variant biology: immune escape, transmission and fitness. Nat Rev Microbiol 2023; 21:162–177. 36653446 10.1038/s41579-022-00841-7 PMC9847462

[10] Weissman D Alameh M-G de Silva T. D614G spike mutation increases SARS CoV-2 susceptibility to neutralization. Cell Host Microbe 2021; 29:23–31. e4. 33306985 10.1016/j.chom.2020.11.012 PMC7707640

[11] Rees-Spear C Muir L Griffith SA. The effect of spike mutations on SARS-CoV-2 neutralization. Cell Rep 2021; 34:108890. 33713594 10.1016/j.celrep.2021.108890 PMC7936541

[12] Islam MA. A review of SARS-CoV-2 variants and vaccines: viral properties, mutations, vaccine efficacy, and safety. Infect Med (Beijing) 2023; 2:247–261. 38205179 10.1016/j.imj.2023.08.005 PMC10774670

[13] Vashisht V Vashisht A Mondal AK. Genomics for emerging pathogen identification and monitoring: prospects and obstacles. BioMedInformatics 2023; 3:1145–1177.

[14] Popa A Genger J-W Nicholson MD. Genomic epidemiology of superspreading events in Austria reveals mutational dynamics and transmission properties of SARS-CoV-2. Sci Transl Med 2020; 12:eabe2555. 33229462 10.1126/scitranslmed.abe2555 PMC7857414

[15] Koopsen J van Ewijk CE Bavalia R. Epidemiologic and genomic analysis of SARS-CoV-2 delta variant superspreading event in nightclub, the Netherlands, June. Emerg Infect Dis 2022; 28:1012–1016. 35271792 10.3201/eid2805.212019 PMC9045423

[16] Lemieux JE Siddle KJ Shaw BM. Phylogenetic analysis of SARS-CoV-2 in Boston highlights the impact of superspreading events. Science 2021; 371:eabe3261. 33303686 10.1126/science.abe3261 PMC7857412

[17] Berno G Fabeni L Matusali G. SARS-CoV-2 variants identification: overview of molecular existing methods. Pathogens 2022; 11:1058. 36145490 10.3390/pathogens11091058 PMC9504725

[18] WHO. Genomic sequencing of SARS-CoV-2: a guide to implementation for maximum impact on public health. World Health Organization; 8 January 2021. https://www.who.int/publications/i/item/9789240018440. [Accessed March 25, 2025].

[19] World Health Organization. Recommendations for national SARS-CoV-2 testing strategies and diagnostic capacities: interim guidance, 25 June 2021. World Health Organization; 2021. Report No.: WHO/2019-nCoV/lab_testing/2021.1. https://apps.who.int/iris/handle/10665/342002. [Accessed March 25, 2025].

[20] Zhang H Jain C Aluru S. A comprehensive evaluation of long read error correction methods. BMC Genomics 2020; 21: 10.1186/s12864-020-07227-0 PMC7751105 33349243

[21] Ostfeld RS Glass GE Keesing F. Spatial epidemiology: an emerging (or re-emerging) discipline. Trends Ecol Evol 2005; 20:328–336. 16701389 10.1016/j.tree.2005.03.009

[22] Elliott P Wartenberg D. Spatial epidemiology: current approaches and future challenges. Environ Health Perspect 2004; 112:998–1006. 15198920 10.1289/ehp.6735 PMC1247193

[23] Goel V Emch M. Spatial epidemiology: challenges and methods in COVID-19 research. COVID-19 and similar futures. 2021; Cham: Springer International Publishing, 23–29.

[24] Snow J. On the mode of communication of cholera. 1855. https://books.google.ch/books?hl=ko&lr=&id=-N0_AAAAcAAJ&oi=fnd&pg=PP12&dq=On+the+Mode+of+Communication+of+Cholera.&ots=mYMkCoMxHO&sig=bd7y7Br6K2O1iYq18qVfs0rhKig. 2053025

[25] Natural Nidality of Transmissible Diseases: With Special Reference to the Landscape Epidemiology of Zooanthroponoses.

[26] Beale L Abellan JJ Hodgson S Jarup L. Methodologic issues and approaches to spatial epidemiology. Environ Health Perspect 2008; 116:1105–1110. 18709139 10.1289/ehp.10816 PMC2516558

[27] Proqio Blogs. [cited 17 October 2024]. https://www.proqio.com/blog/understanding-gis-layers.

[28] Kirby RS Delmelle E Eberth JM. Advances in spatial epidemiology and geographic information systems. Ann Epidemiol 2017; 27:1–9. 28081893 10.1016/j.annepidem.2016.12.001

[29] Race. Gender, and monitoring Socioeconomic gradients in health: a comparison of area-based socioeconomic measures-The Public Health Disparities Geocoding Project. 10.2105/ajph.93.10.1655 PMC1448030 14534218

[30] Shrestha S Stopka TJ. Spatial epidemiology and public health. Geospatial technology for human well being and health. 2022; Cham: Springer International Publishing, pp. 49–77.

[31] What is geocoding and why is it important? - Mapbox. [cited 18 October 2024]. https://www.mapbox.com/insights/geocoding.

[32] Elliott P, Wakefield JC, Best NG, Briggs DJ. Spatial epidemiology: methods and applications. In: Elliott P, Wakefield JC, Best NG, Briggs DJ, editors. Spatial Epidemiology. Oxford: Oxford University Press; 2001. pp. 3–14.

[33] Precision public health and precision medicine: two peas in a pod. [cited 18 November 2024]. https://blogs.cdc.gov/genomics/2015/03/02/precision-public/.

[34] Khoury MJ Bowen MS Clyne M. From public health genomics to precision public health: a 20-year journey. Genet Med 2018; 20:574–582. 29240076 10.1038/gim.2017.211 PMC6384815

[35] Khoury MJ Holt KE. The impact of genomics on precision public health: beyond the pandemic. Genome Med 2021; 13:67. 33892793 10.1186/s13073-021-00886-y PMC8063188

[36] Attwood SW Hill SC Aanensen DM. Phylogenetic and phylodynamic approaches to understanding and combating the early SARS-CoV-2 pandemic. Nat Rev Genet 2022; 23:547–562. 35459859 10.1038/s41576-022-00483-8 PMC9028907

[37] Cromley EK. Using GIS to address epidemiologic research questions. Curr Epidemiol Rep 2019; 6:162–173.

[38▪] Choi Y Ladoy A De Ridder D. Detection of SARS-CoV-2 infection clusters: the useful combination of spatiotemporal clustering and genomic analyses. Front Public Health 2022; 10:1016169. 36568782 10.3389/fpubh.2022.1016169 PMC9771593

[39] Shi Q Herbert C Ward DV. COVID-19 variant surveillance and social determinants in central Massachusetts: development study. JMIR Form Res 2022; 6:e37858. 35658093 10.2196/37858 PMC9196873

[40▪] Woo HD Song DS Choi SH. Integrated dataset of the Korean Genome and Epidemiology Study cohort with estimated air pollution data. Epidemiol Health 2022; 44:e2022071. 36108673 10.4178/epih.e2022071 PMC9849844

[41] Giovanetti M Slavov SN Fonseca V. Genomic epidemiology of the SARS-CoV-2 epidemic in Brazil. Nat Microbiol 2022; 7:1490–1500. 35982313 10.1038/s41564-022-01191-z PMC9417986

[42] Alteri C Cento V Piralla A. Genomic epidemiology of SARS-CoV-2 reveals multiple lineages and early spread of SARS-CoV-2 infections in Lombardy. Italy Nat Commun 2021; 12:434. 33469026 10.1038/s41467-020-20688-x PMC7815831

[43] Hill V Ruis C Bajaj S. Progress and challenges in virus genomic epidemiology. Trends Parasitol 2021; 37:1038–1049. 34620561 10.1016/j.pt.2021.08.007

[44] Geoghegan JL Ren X Storey M. Genomic epidemiology reveals transmission patterns and dynamics of SARS-CoV-2 in Aotearoa New Zealand. Nat Commun 2020; 11:6351. 33311501 10.1038/s41467-020-20235-8 PMC7733492

[45] Preparedness P. Global genomic surveillance strategy for pathogens with pandemic and epidemic potential, 2022–2032. World Health Organization; 28 March 2022 [cited 2 October 2024]. https://www.who.int/publications/i/item/9789240046979. 10.2471/BLT.22.288220 PMC8958828 35386562

